# Trichohepatoenteric Syndrome or Syndromic Diarrhea—Report of Three Members in a Family, First Report from Iran

**DOI:** 10.1155/2016/9684910

**Published:** 2016-01-06

**Authors:** F. E. Mahjoub, F. Imanzadeh, S. Mahdavi Izadi, A. Nahali Moghaddam

**Affiliations:** ^1^Imam Khomeini Hospital Complex, Tehran University of Medical Sciences, Tehran 14197-33141, Iran; ^2^Maternal, Fetal and Neonatal Research Center, Tehran University of Medical Sciences, Tehran 14197-33141, Iran; ^3^Pediatric Nephrology Research Center, Tehran University of Medical Sciences, Tehran 14197-33151, Iran; ^4^Roshan Azma Pathobiology Private Laboratory, Tehran 15149-35754, Iran; ^5^Pediatric Gastroenterology, Mofid Children Hospital, Shahid Beheshti University of Medical Sciences, Tehran 15514-15468, Iran

## Abstract

*Introduction.* Intractable diarrhea of infancy (IDI) includes several types of early onset diarrhea; one of the rare etiologies is trichohepatoenteric (THE) syndrome, also known as syndromic diarrhea (SD) which was primarily described by Stankler et al. Hereby we report a family with several affected members which to our knowledge is the first case report from Iran.* Report of Cases.* A three-year-old boy referred with short stature, poor weight gain, and intermittent steatotic diarrhea to our center. He was born to healthy, relative parents (cousins). He did not gain any weight after four months of age and began having intermittent steatotic diarrhea, abdominal distension, and fever. He was hospitalized several times. Two other children in the family also showed somewhat similar symptoms. Two sweat tests were negative for cystic fibrosis. Workup for Celiac disease was performed several times which was negative; however, gluten-free diet was tried several times which was not effective. Workup for Hirschsprung's disease was performed but colon was ganglionic. Evidence of liver involvement was approved by elevated liver enzymes and coarse echo of liver on sonography.* Discussion.* Trichoenterohepatic syndrome should be put in mind in cases of intractable diarrhea presenting in a family with several affected members. Early diagnosis would save patients from unnecessary workups.

## 1. Introduction

Diarrhea is a common disease among children and constitutes a major etiology for their morbidity and mortality, so understanding different pathologies that cause diarrhea is of importance [1]. Intractable diarrhea of infancy (IDI) is a term which includes several types of early onset diarrhea, one of the rarest is trichohepatoenteric (THE) syndrome, also known as syndromic diarrhea (SD). THE syndrome was primarily described by Stankler et al. in 1982, in two siblings, presenting with diarrhea, failure to thrive, abnormal facies, and unusual hair [2]. Following reports on other cases revealed similarities in signs and symptoms. The most common observed signs are intractable diarrhea, usually starting before six months of age, dysmorphic facies such as prominent forehead and cheeks, broad nasal root and hypertelorism, abnormal hair, disorders of immune system, intrauterine growth retardation, skin abnormalities, and liver function disorders [1, 3, 4]. Distinct hair abnormalities such as woolly, easily removable hair and poorly pigmented hairs are seen even in children with middle eastern origin [3]. Microscopic examination of hair shafts reveals nonspecific abnormalities such as twisted hair (pili torti), aniso- and poikilotrichosis, trichorrhexis nodosa, and longitudinal breaks [3]. Even with treatment, most patients have a small final stature [3–5]. Fine motor movement abnormalities and mental retardation are seen in most affected children [3]. Liver disease (liver enlargement and cirrhosis) is reported in more than half of the children [1]. Investigations show the association between mutations in TTC37 (encodes the putative protein thespin) and SKIV2L with THE syndrome [1, 4, 6, 7]. The pattern of transmission is autosomal recessive [3]. Diagnosis is mainly based on observing different clinical signs, usually in a patient presenting with diarrhea. Intestinal biopsies are more useful for ruling out differential diagnosis of intractable diarrhea than to confirm the diagnosis of THE syndrome, since there are no specific changes related to this disease [1, 3]. Most patients need parenteral nutrition and do not reach their optimal growth eventually [3, 8].

There are also few reports about patients with hemochromatosis of liver associated with this syndrome [9, 10].

Definite diagnosis of the disease is problematic due to variable clinical presentations, nondiagnostic changes in pathology samples, and general lack of an accessible diagnostic test [6, 9].

## 2. Report of the Cases

**Figure 1:**
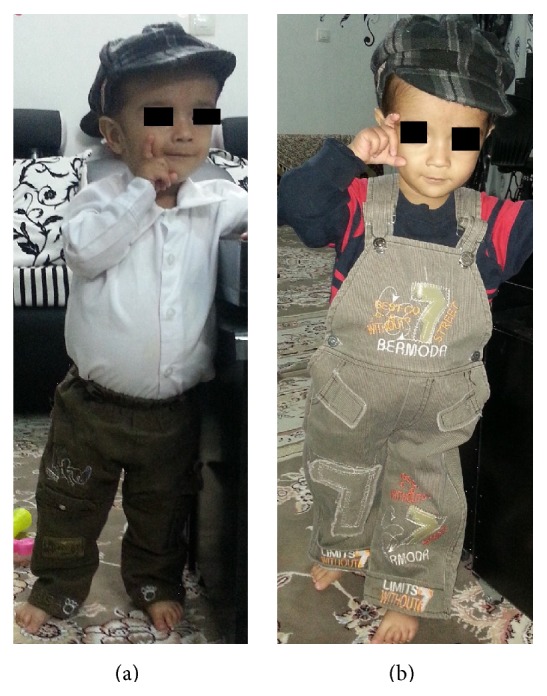
(a) and (b) Note the prominent forehead and cheeks, hypertelorism and broad nasal root, short stature, and abdominal distention of the child.

**Figure 2:**
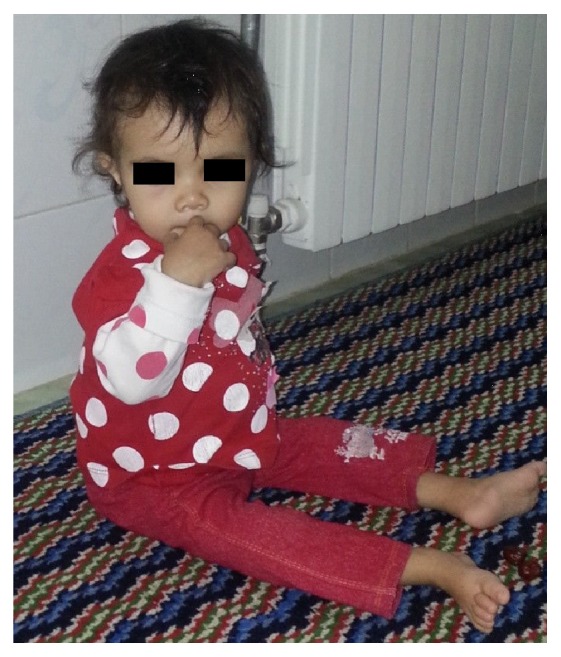
A one-year-old girl with failure to thrive and motor delay. Note facial dysmorphism (hypertelorism and broad nasal root).

**Figure 3:**
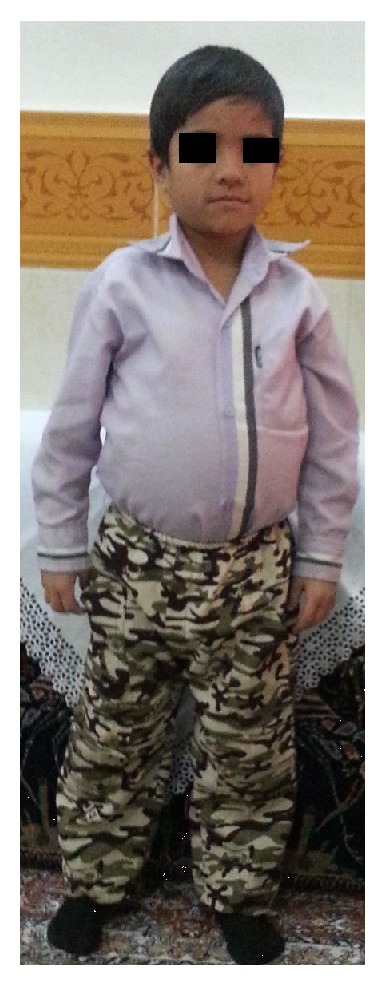
A nine-year-old boy with failure to thrive and abdominal distention. Note facial dysmorphism (hypertelorism and broad nasal root).

A three-year-old boy was born to healthy, related parents (cousins). The pregnancy was uneventful and he was born full term, weighing 3.100 kilograms, and 51 cm in height. He was breastfed and experienced normal growth for the first 4 months and reached 5 kilograms. He had poor weight gain afterwards (recent weight: 8.5 Kg) and began having intermittent steatotic diarrhea and abdominal distension and was hospitalized several times for workup of failure to thrive. As seen in the attached photo facial dysmorphism (prominent forehead, broad based nasal bridge, and hypertelorism) is evident (Figures 1(a) and 1(b)). Hair changes such as woolly and easily removable hair and light coloration were evident in our case. Two other children in the family (his cousin a one-year-old girl weighing 7 Kg with developmental delay and another cousin a 9-year-old boy weighing 16 Kg) also showed somewhat similar symptoms (Figures 2 and 3). Pictures were provided by the family and they were informed about writing of this paper and the paper was submitted after their consent for publishing the pictures with covered eyes.

**Figure 4:**
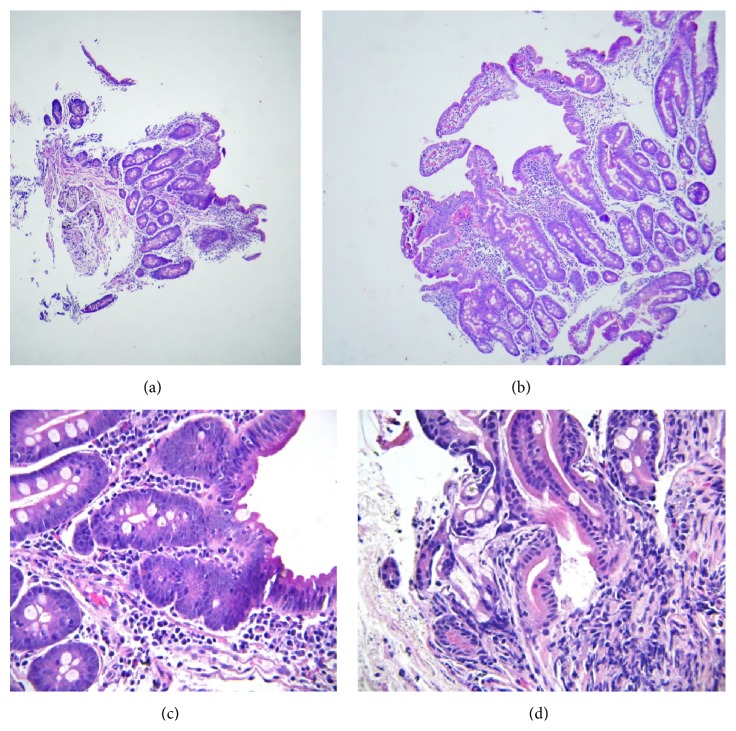
Duodenal mucosa: (a) and (b) moderate to severe shortening of most villi (few rather tall villi are seen in picture (b)). (c) Focal superficial epithelial changes (poor gobletting, irregularity of lining cells) (intraepithelial lymphocytes: under 10/100). There is also mild to moderate infiltration of lymphoplasma cells and some eosinophils (3–7/100) in lamina propria. (d) Note crypt architectural changes.

Several stool exams were performed and were negative for ova, parasites, white blood cells, and red blood cells but were intermittently positive for fat. Stool trypsin activity test was normal. Complete blood cell count (CBC) constantly showed microcytic anemia. Two sweat tests were negative for cystic fibrosis. Multiple testing for Anti-TTG showed variable results (mostly revealed mild elevation). Gluten-free diet was instituted several times but was not effective. Human Leukocyte Antigen (HLA) typing for DQ2 and DQ8 was negative. Workup for Hirschsprung's disease (due to abdominal distention) was performed at 21 months of age, but colon was ganglionic. Thyroid function tests were normal. Insulin Growth Factor 1 (IGF-1) was lower than normal in one occasion (7.34 ng/mL, normal: 27.4–113.5 ng/mL). Abdominal sonography at 1.5 years of age showed coarse and nonhomogeneous echo of liver. AST and ALT were mildly elevated. Upper gastrointestinal endoscopy showed evidence of esophagitis and mild atrophic duodenitis. Biopsy from esophagus and mucosa of second part of duodenum was performed. Microscopic examination of esophageal mucosa showed moderate esophagitis. In duodenal mucosa, the villi were mildly to moderately short in well oriented areas. Focal superficial epithelial changes were seen (reduced number of goblet cells, irregularity of lining cells) (Intraepithelial lymphocytes: under 10/100). Crypt hyperplasia cannot be assessed; however, crypts show marked difference in size, branching, and distortion. There was mild to moderate infiltration of lymphoplasma cells and some eosinophils (3–7/100) in lamina propria, distorting some of the crypts. No giardia lamblia parasites are seen (Figure 4).

**Figure 5:**
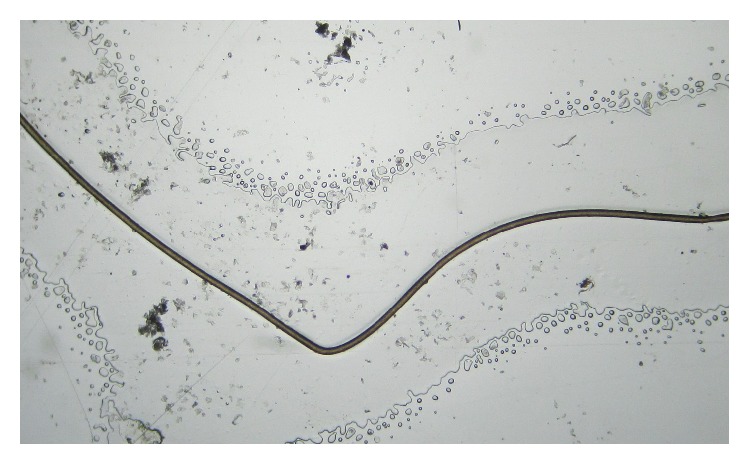
Hair shaft of the child showing kinking.

Microscopic examination of hair shafts of the first case revealed aniso- and poikilotrichosis with kinking and breakage but no typical trichorrhexis nodosa was seen (Figure 5).

Hair changes in cases 2 and 3 are mild and not as prominent as case 1, which denotes that spectrum of changes is seen in the family. Also the index patient suffers from severe dental problems (caries) leading to loss of most teeth. No skin or cardiac involvement is present in patients.

No further workup for liver disease and liver biopsy was performed for the patient.

The case was consulted with Professor Elizabeth Montgomery (Johns Hopkins School of Medicine, USA) and, in light of clinical symptoms (facial dysmorphism, hair anomaly, and hepatic involvement along with gastrointestinal symptoms) and affection of several family members, diagnosis of trichohepatoenteric syndrome was established. Genetic testing is not available yet in Iran. Diagnosis was accepted readily by pediatric gastroenterologists and the patient was relieved from further repetitive and time consuming workup tests.

## 3. Discussion

Despite high prevalence of diarrhea in children and availability of several tests for investigating different etiologies, still in many cases, even with extensive investigations, definite diagnosis cannot be reached. Moreover we are yet far from developing a way to control the etiology in many cases. Trichoenterohepatic syndrome was first described by Stankler et al. in 1982 [2]. Since then several cases have been reported and researchers have concluded that the diagnosis is mainly suspected by clinical signs and symptoms such as persistent diarrhea, facial dysmorphism, abnormal hair, and involvement of liver. To date the confirmatory diagnostic test is sequencing of TTC37 and SKIV2L genes [1, 4, 7] and microscopic examination of intestinal biopsies is more useful in ruling out other causes of chronic diarrhea such as tufting enteropathy and congenital microvillus atrophy. Treatment of these children is mainly symptom-relieving actions such as parenteral nutrition to help with growth, sometimes immunoglobulin supplementation, and, in those with severe liver disease, liver transplant [1]. Even with treatment, most patients have a small final stature [3–5]. About half of them show a slight mental retardation, but the main observed complications are liver disease and infections. Understanding the genetic defect of the disease gives hope to better perception of the pathology of the disease and perhaps a better management of patients.

Diagnosis was delayed for several years in our patient and he underwent unnecessary workups such as biopsy of colonic wall and testing for celiac disease several times. A careful clinical examination and thorough asking about family history (presence of affected members, consanguinity) would have helped to reach to a diagnosis sooner and save patient from time consuming and invasive workups.

Although genetic testing is the definite diagnostic test, unfortunately it is not yet available for this syndrome in our country; however, the symptoms and family history leave no doubt about the diagnosis.

Our cases showed no evidence of overt mental retardation which is seen in some cases although motor delay was seen in the one-year-old girl mentioned.
